# Biodegradation of Silk Biomaterials

**DOI:** 10.3390/ijms10041514

**Published:** 2009-03-31

**Authors:** Yang Cao, Bochu Wang

**Affiliations:** 1 College of Biological and Environmental Engineering, Jiangsu University of Science and Technology, Zhenjiang Jiangsu 212018, P.R. China; E-Mail: bestmancy@163.com; 2 College of Bioengineering, Chongqing University, Chongqing 400030, P.R. China

**Keywords:** Biodegradation, Silk Biomaterials, Enzymatic Degradation

## Abstract

Silk fibroin from the silkworm, *Bombyx mori*, has excellent properties such as biocompatibility, biodegradation, non-toxicity, adsorption properties, etc. As a kind of ideal biomaterial, silk fibroin has been widely used since it was first utilized for sutures a long time ago. The degradation behavior of silk biomaterials is obviously important for medical applications. This article will focus on silk-based biomaterials and review the degradation behaviors of silk materials.

## 1. Introduction

What is a biomaterial? Biomaterial can be defined as “any substance (other than a drug) or combination of substances synthetic or natural in origin, which can be used any time, as a whole or as a part of a system which treats, augments, or replaces any tissue, organ or function of the body”[1]. Theoretically, any material, natural or man-made, can be a biomaterial as long as it serves the stated medical and surgical purposes. The development of biomaterials is not a new area. It encompasses elements of medicine, biology, physical, chemistry, tissue engineering and materials science. Nevertheless, the demand for biocompatible, biodegradable and bioresorbable materials has increased dramatically since the last decade. An ideal biomaterial is one that is non-immunogenic, biocompatible and biodegradable, which can be functionalized with bioactive proteins and chemicals. In particular, biodegradability is one of the essential properties of the biomaterials. Over the past decades, significant attention has been paid to the biodegradable biomaterials. Here are some of the important properties of biodegradable biomaterials [2]:

- The material should not evoke a sustained inflammatory or toxic response upon implantation *in vivo*.
- The material should have acceptable shelf life.
- The degradation time of the material should match the healing or regeneration process.
- The material should have appropriate mechanical properties for the indicated application, and any variation in mechanical properties with degradation should be compatible with the healing or regeneration process.
- The degradation products should be non-toxic, and easily metabolized and cleared from the body.
- The material should have appropriate permeability and processibility for the intended application.

Consequently, a wide variety of natural and synthetic biodegradable polymers have been investigated recently for medical and pharmaceutical applications. Natural biodegradable polymers like collagen, gelatin, chitosan and silk fibroin have promising advantages over synthetic polymers due to their favorable properties, including good biocompatibility, biodegradability and bioresorbability. Their physical and chemical properties can be easily modified to achieve desirable mechanical and degradation characteristics. Among these natural polymers, silk fibroin provides an important set of material options for biomaterials and scaffolds in biomedical applications because of its high tensile strength, controllable biodegradability, haemostatic properties, non-cytotoxicity, low antigenicity and noninflammatory characteristics [3–5].

Silk fibroin is a natural protein produced by the domestic silkworm, *Bombyx mori*. It can be used as a biomaterial in various forms [6], such as films [7–9], membranes [10], gels [11], sponges [12], powders [13], and scaffolds [14–16]. Applications include burn-wound dressings [17], enzyme immobilization matrices [18], nets [19], vascular prostheses and structural implants [20–21]. Silk has been commercially used as biomedical sutures since decades of years ago. Because of its special crystallization and orientation, as well as compact structure, natural fibroin is difficult to degrade. As a kind of FDA approved biomaterial, silk is defined by United States Pharmacopeia as non-degradable for its negligible tensile strength loss *in vivo*. However, according to the literature, silk is degradable but over longer time period. In general, silk is slowly absorbed *in vivo*. The rate of degradation depends on many factors. This article will focus on the silk-based biomaterials, and review the degradation behaviors of silk materials.

## 2. Structure and Properties of Silk Biomaterials

The silk worm has been domesticated for thousands of years. The cocoon is wrapped in a continuous silk thread whose length can exceed 1 km [22]. Normally, native silk fiber consists of two types of self-assembled proteins: fibroin and sericin [23,24]. These two proteins both contain the same 18 amino acids such as glycine, alanine and serine in different amounts. The core fibroins are encased in a coat of sericin, a family of hydrophilic proteins which holds two fibroin fibers together [25–26]. There is a kind of proteins that non-covalently linked these proteins named P25, a 25 kDa glycoprotein [24,27]. The fibroin is a giant molecule comprising a crystalline portion of about two-thirds and an amorphous region of about one-third. The crystalline portion contains repetitive amino acids (-Gly-Ala-Gly-Ala-Gly-Ser-) along its sequence, forming an antiparallel β-sheet and leading to the stability and mechanical properties of the fiber [22,28–30].

Generally, the main secondary structures of fibroin are of the random-coil and amorphous type and the antiparallel β-sheet type, which is formed through hydrogen bonds between adjacent peptide chains [31]. The silk fibroins are characterized as natural block copolymers comprising hydrophobic blocks with short side-chain amino acids such as glycine and alanine, and hydrophilic blocks with larger side-chain amino acids, as well as charged amino acids [32]. The former blocks lead to β-sheets or crystals through hydrogen bonding [14]. The two main distinct structures in silk fibroin are silk I and silk II. The structure of silk I contains random-coil and amorphous regions. The silk II structural form of the silk fibroins has been characterized as an antiparallel β-sheet structure. The former structure is a water-soluble structure while the latter excludes water and is insoluble in several solvents including mild acid and alkaline conditions, and several chaotropes [33] (Table 1). In regenerated silk fibroins, the silk I structure easily converts to a β-sheet structure by chemical methods such as treatment with methanol [34–36].

Compared to other biomaterials, silk fibroins have excellent mechanical properties such as remarkable strength and toughness (Table 2). In the final molecular assembly of the proteins into silk fibers, the hydrophobic domains play an important role [35]. These domains take up a large portion of silk fibroin and are responsible for insolubility, the high strength of fibers and the thermal stability of the silk fibers which lead to the formation of β-sheet secondary structure [32]. It can be concluded that the materials properties of silk fibroins such as biodegradability and biocompatibility, are determined by their special molecular structure [35].

## 3. Degradation Behaviors of Silk Biomaterials

According to the US Pharmacopeia’s definition, silk is classified as non-degradable. However, from the literature, it can be considered as a degradable material. The reason may be connected to the fact that silk degradation behavior is usually mediated by a foreign body response [26,41–43]. Different from synthetic materials, the degradable behavior of silk fibroins doesn’t lead to an immunogenic response. Biodegradation is the breakdown of polymer materials into smaller compounds. The processes vary greatly, and the mechanisms are complex. Normally, the encompass physical, chemistry and biological factors. Depending on the mode of degradation, silk fibroins can be classified as enzymatically degradable polymers [44,45]. Enzymes play a significant role in the degradation of silk fibroins. Due to their enzymatic degradability, unique physic-chemical, mechanical and biological properties of silk fibroins have been extensively investigated. The enzymatic degradation of biomaterials is a two-step process. The first step is adsorption of the enzyme on the surface of the substrate through surface-binding domain and the second step is hydrolysis of the ester bond [45].

### 3.1. The biodegradation behavior of silk biomaterials with sifferent enzymes

As a protein, silk fibroin is susceptible to biological degradation by proteolytic enzymes such as chymotrypsin, actinase, and carboxylase [12,46–48]. Generally, the biodegradation behavior has two steps, as explained above. At first, silk biomaterials are adsorbed by different enzymes, which demands that the enzymes must find binding domains on the materials’ surface. After that, silk biomaterials are digested by enzymes. The final wastes of silk fibroins are the corresponding amino acids, which are easily absorbed *in vivo*. That is one of advantages of silk biomaterials used in biomedical field.

The characteristics of silk biodegradation behaviors vary with different enzymes. Some literature has investigated the degradation behaviors of silk fibroins exposed to different proteolytic enzymes for various times. Chymotrypsin has been used to degrade amorphous regions of fibroins to obtain highly crystallizable fibroin protein [12]. When protease (Protease XIV) was compared with α-chymotrypsin, silk matrices incubated in the former enzyme significantly decreased in mass and UTS a week later, while, when in α-chymotrypsin, the UTS and mass of the silk matrices remained unchanged [49]. In another way, Li and his team [12] find α-chymotrypsin could degrade the dissolved fibroin proteins but not the fibroin sheet. In contrast, other enzymes (particularly protease XIV) extensively degraded the fibroin sheets demonstrating the potential of protease degradation of silk fibroin.

After biodegradation, significant changes have been reported according to the structure and molecular weight of silk fibroins. In *in vitro* studies of silk degradation behavior with proteolytic enzymes they will cleave the less-crystalline regions of the protein to peptides which are then capable of being phagocytosed for further metabolism by the cell [26,44]. In one study [10], protease E degraded the surface of silk fibroin membranes, especially the amorphous regions. Generally, degradation behavior varies from different silk materials forms with regard to enzymes. From other literature, when a silk fibroin sheet is immersed in various proteolytic enzyme solutions, the small amount of Silk II crystalline structure originally present in the sheet will disappear after degradation by protease XIV, and Silk I crystalline structure formed leading to an overall increase in crystallinity of the silk film over time. Collagenase IA is shown to degrade silk II as well, but to a lesser extent. α-Chymotrypsin is believed to degrade silk [44,50], however, it does not have an appreciable effect on the degradation of silk films [12,49].

In another way, biodegradation behavior has great effect on the final molecular weight after degradation. Upon incubation with proteolytic enzymes, silk films exhibit a noticeable decrease of sample weight and degree of polymerization to an extent which depended on the type of enzymes, on the enzyme-to-substrate ratio, and on the degradation time [44]. Focusing on three types of enzymes as examples, protease was more aggressive than α-chymotrypsin or collagenase. The average molecular weight of silk biomaterials after degradation follows the order protease XIV < collagenase IA < α-chymotrypsin [12].

From the literature, the changes in sample weight and degree of polymerization of silk fibers exposed to proteolytic attack are negligible. However, tensile properties are also significantly affected, as shown by the drop of strength and elongation as a function of the degradation time [44]. Silk can be proteolytically degraded and resorbed *in vivo* over a longer time period (typically within a year) [31,49]. *In vivo* studies, silks lose the majority of their tensile strength within one year, and fail to be recognized at the site within two years or even longer [25]. Great changes have happened to the morphology of silk fibroin, such as diameter, strength, and surface roughness. Enzymes, such as protease XXI, have been shown to degrade silk films and fibers altering surface roughness and strength over 17 days [42]. In order to know the biodegradation behavior of silk fibroin, several studies implanted silk materials under the skin of rats *in vivo*. After 6 weeks post-implantation, 55% of silk tensile strength and 16% of elastic modulus were found to be lost [51–52]. In another rat model, silk fibers lost 29% of tensile strength at 10 days, 73% at 30 days and 83% after 70 days [52]. Another study regarding molecular-weight distribution and amino acid composition indicates that part of silk fibroin materials is broken down into amino acids [12]. When immersed in collagenase IA, the weight of the fibroin sheets decreases as the degradation time increases. The percentage of free amino acids exceeded 50% of the total. Particularly, 70% of a silk fibroin sheet can be degraded in 15 days when exposed to protease XIV. Importantly the protease XIV does not only degrade silk fibroin, but also directly degraded the fibroin sheet into peptides and amino acids. This indicates that the biodegradation products of silk fibroin materials do less or even no harm to the human body.

### 3.2. Factors influencing the degradation behavior of silk biomaterials

The degradation behavior of biomaterials is important in medical applications *in vivo*. The features of enzymes influence the biodegradable process for silk fibroins. For example, most proteolytic enzymes are better at degrading silk fibroins with low molecular weight and non-compact structures [53]. This indicates that the degradation behavior has a close relationship with the molecular weight and structure of silk biomaterials, so structure and molecular weight of polymers are two main factors influencing the biodegradation process. Low molecular weight and non-compact structure means that it is easy for enzymes to bind on the surface of silks as well as display hydrolysis behaviors.

As for silk fibroin porous sponges, their biodegradation behavior depends on the original preparation method and structural characteristics, such as processing condition, pore size, silk fibroin concentration, and host immune system elements during degradation [54]. It maybe has a close relationship with increased surface roughness or differences in content or distribution of crystallinity [34]. Thus it is possible to regulate the degradation behavior of silk fibroin by changing the crystallinity [10], pore size, porosity and molecular weight distribution of the silk fibroin. Wang’s [54] research was conducted to systematically investigate the degradation behavior of silk fibroin three-dimensional scaffolds in both nude and Lewis rats. The study indicated that the *in vivo* behavior of the silk fibroin scaffolds can be predicted and thus can be controlled to match the diverse needs for the engineering and repairing of various tissues with specific functional requirements, repairing characteristics, and repairing rates.

It is generally accepted that the degradation of silk materials should match the function needs and ensure optimum mechanical and physiological integration of the device. Control over the rate is an important feature of function tissue design, for example the rate of scaffold degradation should match the rate of tissue growth [33]. Based on varieties studies on natural polymers, the rate and extent of degradation may be highly variable, depending on a series of factors related to structural and morphological features of the polymers, processing conditions, as well as characteristics of the biological environment at the location of implantation, and presence of different mechanical and chemical stresses [44]. Some researchers have indicated that variable rates of silk absorption *in vivo* are dependent on the animal model and tissue implantation site (Table 3) [26]. The degradability of silk fibroin also can be altered by processing conditions. Different processing conditions influence silk materials degradability significantly. As an enzymatically degraded biomaterial, the rate of silk degradation partly depends on the availability and concentration of the enzymes. Besides, chemical modification also affects the degradation behavior [33,45].

### 3.3. Others

According to what has been discussed, protease cocktails and chymotrypsin are capable of enzymatically degrading silk [26]. Of interest, the silkworm, *Bombyx mori*, produces a protease inhibitor in the silk gland embedding it within the cocoon for protection against premature proteolytic degradation [57]. Generally, this 6kDa trypsin inhibitor is isolated from the water extract from silkworm cocoons, and protects the light chain of silk fibroin against tryptic degradation.

In addition to proteolytic enzymes, silk fibroins also can be degradated by other means, such as gamma radiation. Gamma radiation directly affects the decreasing tensile strength of the fibroin fibers. Due to the weakness of peptide bonding in fibroin’s polypeptides, reduction of β-sheet structure in the silk fibroin, as well as the release of low-molecular-weight proteins in degradation products, the results of this study shows that the biodegradation of silk fibroin increases with increasing irradiation intensity [58].

## 4. Conclusions

Generally, biodegradability is one of the essential properties of the biomaterials. Over the past decades, significant attention has been paid to biodegradable biomaterials. Silk fibroins are obtained from the cocoons of mulberry silkworm *Bombyx mori*. Due to their high tensile strength, controllable biodegradability, haemostatic properties, non-cytotoxicity, low antigenicity and non-inflammatory characteristics, silk materials are increasingly used as biodegradable material.

Normally, silk fiber consists of two types of self-assembled proteins, fibroin and sericin. The fibroin is a major component of silk fiber serving as the core, while the sericin is a minor component serving as a coating protein. The former is comprised of highly organized β-sheet crystal regions and semi-crystalline regions responsible for silk’s elasticity compared to fibers of similar tensile integrity [26]. Biodegradable materials are preferred candidates for developing therapeutic devices such as temporary prostheses, three-dimensional porous structures as scaffolds for tissue engineering and as controlled/sustained release drug delivery vehicles. The medical application demands biomaterial with special properties such as degradability. According to the US Pharmacopeia’s definition, however, silk is classified as non-degradable. Although according to the literature, it can be considered as a degradable material, although over longer times. Depending on the mode of degradation, silk fibroins can be classified as enzymatically degradable polymers [45]. Enzymes play a significant role in the degradation of silk fibroins, especially proteolytic enzymes. The degradation behavior of biomaterials is important in the medical application *in vivo*. Control over the rate is an important feature of function tissue design, such as the rate of scaffold degradation matches the rate of tissue growth. Based on varieties studies on natural polymers, the rate and extent of degradation may be highly variable, depending on a series of factors related to structural and morphological features of the polymers, such as fibers, films, sponges, processing conditions, as well as characteristics of the biological environment at the location of implantation, and presence of different mechanical and chemical stresses [44]. Moreover, during the biodegradation, some other factors influence the silk biodegradation behavior, such as a protease inhibitor, produced by silk itself, and gamma radiation. Finally, a better understanding of the biodegradation behavior of silk fibers will provide greater insight into the appropriate silk biomaterials design for future medical appliaction.

## Figures and Tables

**Table 1.** Structure of silk fibers.

*Bombyx mori* silk worm				
Silk fiber	Silk fibroin (72–81%)			Silk sericin (19–58%)
	H chain	L chain	P 25 glycoprotein	a glue-like protein
Molecular Weight	325 kDa	25 kDa	25 kDa	~300 kDa
Polarity	Hydrophobic			Hydrophilic
Structure	silk I(random-coil or unordered structure) silk II(crystalline structure) silk III (unstable structure)			non-crystalline structure
Function	the structure protein of fibers filament core protein			binds two fibroins together coating protein

**Table 2.** Mechanical properties of biodegradable materials. Reprinted from [26] Biomaterials, 24 (2003), Gregory H. Altman, Frank Diaz, Caroline Jakuba, Tara Calabro, Rebecca L. Horan, Jingsong Chen, Helen Lu, John Richmond, David L. Kaplan, Silk-based biomaterials, Pages No.401–416, Copyright (2009), with permission from Elsevier.

Source of biomaterial	UTS (MPa)	Modulus (GPa)	Strain (%) at breakage	References
*Bombyx mori* silk (with sericin)	500	5–12	19	[37]
*Bombyx mori* silk (without sericin)	610–690	15–17	4–16	[37]
*Bombyx mori* silk	740	10	20	[38]
Collagen	0.9–7.4	0.0018–0.046	24–68	[39]
Cross-linked collagen	47–72	0.4–0.8	12–16	[39]
Polylactic acid	28–50	1.2–3.0	2–6	[40]

**Table 3.** Evidence of silk degradation *in vitro* and *in vivo*. Reprinted from [26] Biomaterials, 24 (2003), Gregory H. Altman, Frank Diaz, Caroline Jakuba, Tara Calabro, Rebecca L. Horan, Jingsong Chen, Helen Lu, John Richmond, David L. Kaplan, Silk-based biomaterials, Pages No.401–416, Copyright (2009), with permission from Elsevier.

Type of silk	*In vivo/vitro*	Mechanism	Degree and measure of degradation	References
Extracted fibroin film	*In vitro*	Proteolytic degradation	~10% weight loss 5 days following enzymatic digestion	[10]
Unknown/ assumed black raided	Rat/ subcutaneous	Unknown/ assumed foreign body response	55% loss in tensile strength 6 weeks *in vivo*	[51]
Black braided	Rat/ subcutaneous	Unknown/ assumed foreign body response	83% loss in tensile strength 10 weeks *in vivo*	[52]
Unknown/ assumed black raided	Rat/ abdominal wall muscle	Foreign body response (proteolytic degradation)	Fragmentation at 6 weeks; not detected at 24 weeks	[55]
Black braided	Rabbit/ cornea, sclera and ocular muscle	Foreign body response (proteolytic degradation)	Reduced number of filaments and diameter at 42 days; absorption at 90 days *in vivo*	[43]
Unknown/ assumed virgin silk	Rabbit/ abdominal wall muscle	Foreign body response (proteolytic degradation)	80% decrease in tensile strength at 12 weeks; 0% strength at 2 years; decrease in the number of fibers observed histologically; fragmentation following 4 weeks *in vivo*	[56]
